# A dataset of dynamical social map in ancient China: 618−1644

**DOI:** 10.1016/j.dib.2022.108804

**Published:** 2022-12-06

**Authors:** Xiong-Fei Jiang, Ling Bai, Na Zhao, Long Xiong

**Affiliations:** a College of Finance and Information, Ningbo University of Finance and Economics, Ningbo 315175, PR China; b School of Physics and Astronomy, Yunnan University, Kunming 650091, PR China

**Keywords:** Geographic history, Elite, Social networks, Complex networks, Sociophysics

## Abstract

The data set of this article is related to the paper “Dynamical structure of social map in ancient China” (2022)[1]. This article demonstrates the data of social relations between cities in ancient China, ranging from 618 AD to 1644 AD. The raw data of social associations between elites used to build social maps are extracted from the China Biographical Database. The raw data contain 14,610 elites and 29,673 social associations, which cover 366 cities. The dataset of this article is relevant both for social and natural scientists interested in the social and economic history of ancient China. The data can be used for further insights/analyses on the evolutionary pattern of geo-social architecture, and the geo-history from the viewpoint of social network.

**Specifications Table**

**Table:** 

Subject	History
Specific subject area	Sociophysics, Quantitative history, Complex system, Social network, Geographic history
Type of data	Table Figure Interactive HTML
How the data were acquired	The social associations between elites are gathered from the China Biographical Database (CBDB), and the coordinates of cities are downloaded from Baidu Map.
Data format	Raw Analyzed
Description of data collection	We only consider social associations in the CBDB, such as intellectual, literary, political, and other social associations between two individuals, while kinship relations are excluded. Beside, we only consider the elites carrying geographical coordinates, which are the domicile places in the CBDB.
Data source location	Raw data of social associations between elites are extracted from the CBDB, https://projects.iq.harvard.edu/cbdb/home.
Data accessibility	Repository name: Mendeley Data Data identification number: 10.17632/vjyh3g8w2r.2 Direct URL to data: 10.17632/vjyh3g8w2r.2
Related research article	L. Bai, L. Xiong, N. Zhao, K. Xia, X. F. Jiang, Dynamical structure of social map in ancient China, Physica A 607 (2022) 128,209. 10.1016/j.physa.2022.128209.

## Value of the Data

• This dataset presents unique, highly detailed and regionally disaggregated, insights on social associations between elites in ancient China from 618 AD to 1644 AD. This dataset is helpful as it gives a deeper understanding of the organization of cities in ancient China.
• The dataset is relevant both for social and natural scientists (such as historians, economists, geographers, and statistical physicists) interested in ancient China's social and economic history. The figures are particularly valuable for historians working on ancient China.
• The dataset can be introduced for further insights/analyses on the evolutionary pattern of geo-social architecture and the geo-history from the viewpoint of social networks.
• The dataset fits well into the interdisciplinary scope in the sense that we take the complex network approach to tackle historical problems with social relations.

## 1 Objective

The dataset of this article is useful both for social and natural scientists interested in the social and economic history of ancient China. The researchers could further unveil the evolutionary pattern of geo-social architecture via this dataset. For examples, how do the social maps evolve with nations’ borders? What is the connection between the social maps and geographical boundaries? What is the socioeconomic mechanism to account for the formation of social maps? This dataset has various latent applications in geo-history from the viewpoint of social network.

## 2 Data Description

The social structure of elites in premodern empires attracts much attention from multi-disciplines [1], [2], [3], [4], [5]. Meanwhile, the geographic organization of culture and politics is reflected in the organizational structure of cities. In this article, we compile a dataset of the organizational structure of cities named the social map, which is generated from the social associations between elites in ancient China [2].

The dataset contains 3 files: “Networks.xlsx”, “Coordinates.xlsx”, and “SocialMap.html”. The “Networks.xlsx” has 3 columns, representing the source node (city), target node (city), and weight of a link between two nodes, respectively. The weight of a link, which represents the strength of social relation between two cities, is the total number of social connections between two cities. The “Networks.xlsx” contains 9 sheets, which are the data for different dynasties named by Early Tang, Late Tang, Early Northern-Song, Late Northern-Song, Early Southern-Song, Late Southern-Song, Yuan, Early Ming, and Late Ming. In addition, we provide the python code script (generate_social_map.py), which is used to generate the “Networks.xlsx” from the social associations between elites. Noticeably, the “Networks.xlsx” can be visualized by the network software of Gephi directly. The “Coordinates.xlsx” has 4 columns storing longitude and latitude for all cities that appeared in 9 networks. The first and second columns are English names and Chinese names of cities; the third and fourth columns are longitudes and latitudes of cities. The “SocialMap.html” provides a visualization platform, in which users could select and illustrate the evolution of social maps intuitively.

**Fig. 1:**
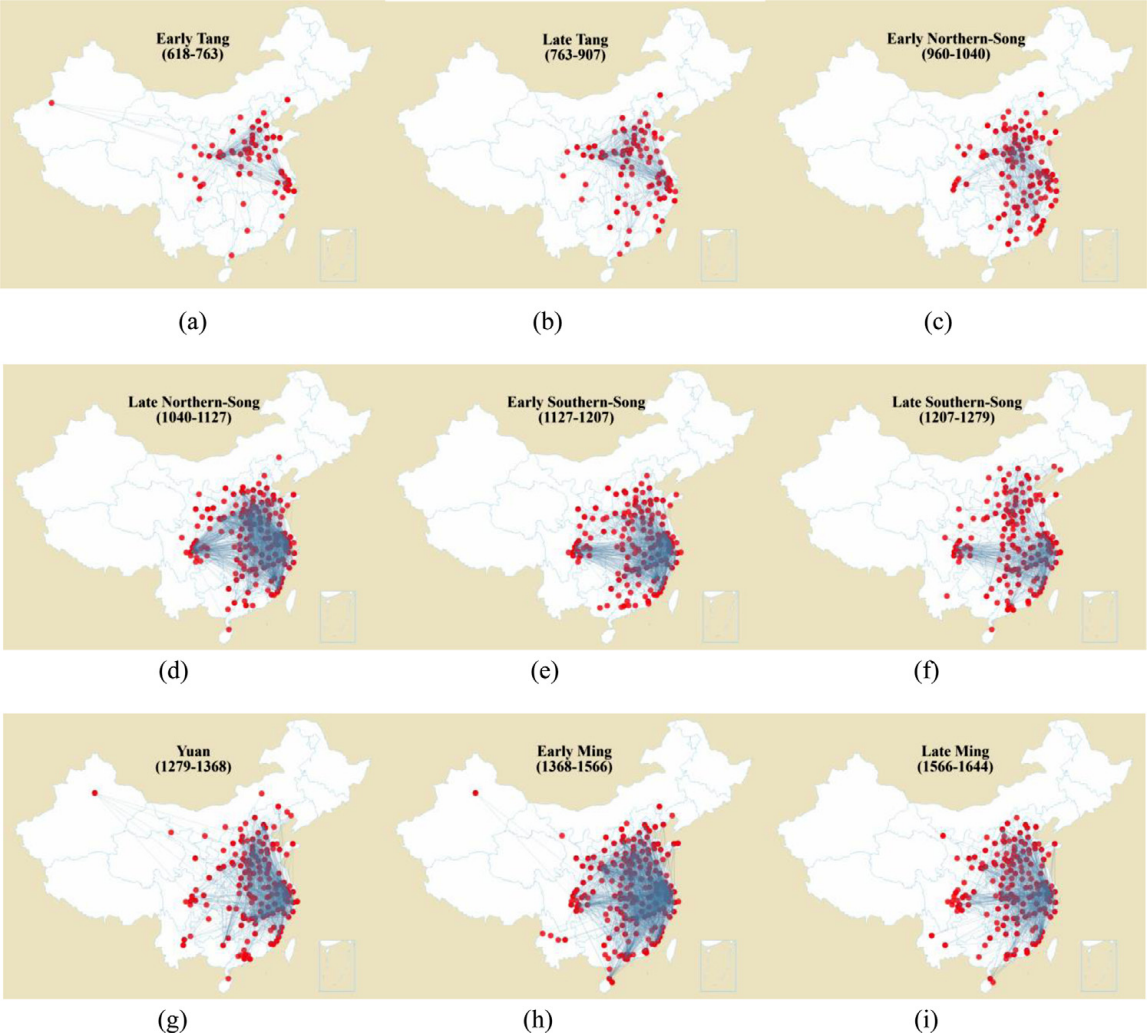
Social maps of cities for every dynasty. All dynasties are divided into the early and late ones, except for the Yuan. For clarity, a social map is embedded in the current map of China, but not the one of the corresponding dynasties.

**Fig. 2:**
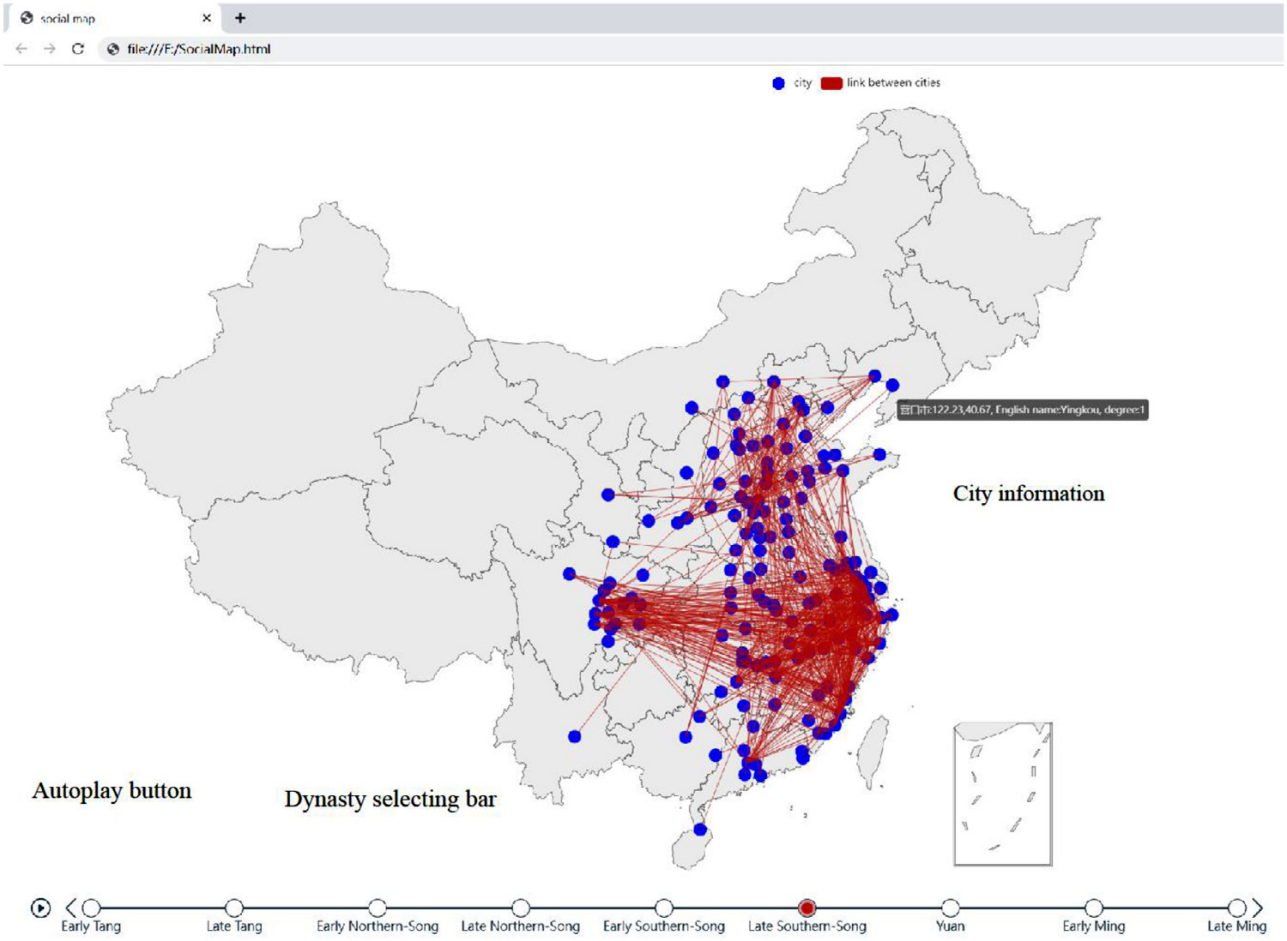
Main page for visualization platform of social maps. This figure is a static demo, while the HTML file is interactive. Cities are denoted by nodes, and provinces are bordered by lines. One could select and compare the social maps by the bar at the bottom. If the mouse is put on a city, the city's name, coordinates, and degree pop up. The social map is autoplayed in default, and can be turned off by the “Autoplay” button.

Table. 1 shows the basic description of data for social maps, ranging from 618 AD to 1644 AD. This period contains the consecutive dynasties, including the Tang, Northern-Song, Southern-Song, Yuan, and Ming. Generally, the size of the social map increases over time. All dynasties maintain about 300 years, except for the Yuan. The Yuan existed for about 150 years. Interestingly, critical events usually occurred in the middle of those dynasties, such as the An-Shi Rebellion in the Tang, and the Qingli Reforming in the Northern-Song. Those events led to tremendous changes in history, and divided every dynasty into two parts. Thus, we classify dynasties into the early and late ones, except for the Yuan. As shown in Fig. 1, the social networks are embedded in the map to demonstrate the geo-social networks intuitively. In addition, Fig. 2 shows the main page of the visualization platform. One can interactively observe the social maps through the bar at the bottom.

**Table 1:** Basic description of the data for the social maps. N and M represent numbers of cities and the number of links between cities, respectively; e and q denote numbers of elites and the number of social associations between elites, respectively. Event is the historical event dividing a dynasty into the early and late ones.

	Year (AD)	*N*	*M*	*e*	*q*	Event
Early Tang	618 - 762	62	228	347	672	An-Shi Rebellion
Late Tang	762 - 907	83	371	498	1090	
Early Northern-Song	960 - 1040	108	406	830	995	Qingli Reforming
Late Northern-Song	1040 - 1127	163	1511	3486	5707	
Early Southern-Song	1127 - 1207	148	1119	3707	5926	Kaixi Northern Expedition
Late Southern-Song	1207 - 1279	148	873	2649	3757	
Yuan	1206 - 1368	155	1167	2748	4911	
Early Ming	1368 - 1566	195	1854	3486	6317	Longqing Opening
Late Ming	1566 - 1644	167	1058	1525	2697

## 3 Experimental Design, Materials and Methods

The raw data in this article comes from the China Biographical Database (CBDB) [6], containing elites and their associations in ancient China. We only consider social associations between two individuals, while kinship relations are excluded. The CDBD determines these non-kinship associations for an individual through historical documents. The CBDB records multiple sorts of non-kinship social associations, with 241 types of associations categorized into 10 classes and 35 subclasses, listed in Table 2. Beside, we only consider the elites carrying geographical coordinates, which are the domicile places. Finally, we collect 14,610 individuals and 29,673 social associations ranging from 618 AD to 1644 AD. This period contains the consecutive dynasties, including the Tang, Northern-Song, Southern-Song, Yuan, and Ming. Notably, if a city uses different names in history, we only quote the current name for all dynasties. For example, although Xi'an is named Chang'an before the Ming, it is referred to as Xi'an for all dynasties in this article.

**Table 2:** The classes and subclasses of the non-kinship social associations in the CBDB.

ID	Class	Subclass
01	Associations (General)	Associations (General)
		Association through common membership
		Social Interactions
02	Scholarship	Teacher-Student
		Intellectual Affiliations
		Association by Scholarly Topic
		Association through common membership
		Academic Patronage
		Literary and Artistic Affiliations
		Intellectual Attacks
03	Friendship	Friendship(General)
04	Politics	Politics (General)
		Connection via office (equal)
		Connection via office (subordinate)
		Connection via office (superior)
		Supportive political association
		Recommendation and sponsorship
		Oppositional political association
05	Writings	Writings (General)
		Commemorative Texts
		Epitaphs
		Prefaces/Postfaces
		Ritual Texts
		Biographical Texts
		Explanatory Texts
		Mottos
		Correspondences
		Occasional Texts
06	Military	Military (General)
07	Medicine	Medicine (General)
08	Religion	Religion (General)
09	Finance	Finance (General)

The CBDB introduces the index year, an arbitrary year assigned to each individual, indicating the peak of one's life. The CBDB has complicated rules to identify the index year. Here we present the rules briefly. Before the 20,190,424 version, the index year is defined as the age of 60. If one passes away before the age of 60, the CBDB uses the death year as the index year. Beside, if an individual's data are missing, his relative's or scholar's year is applied to determine the index year.

**Fig. 3:**
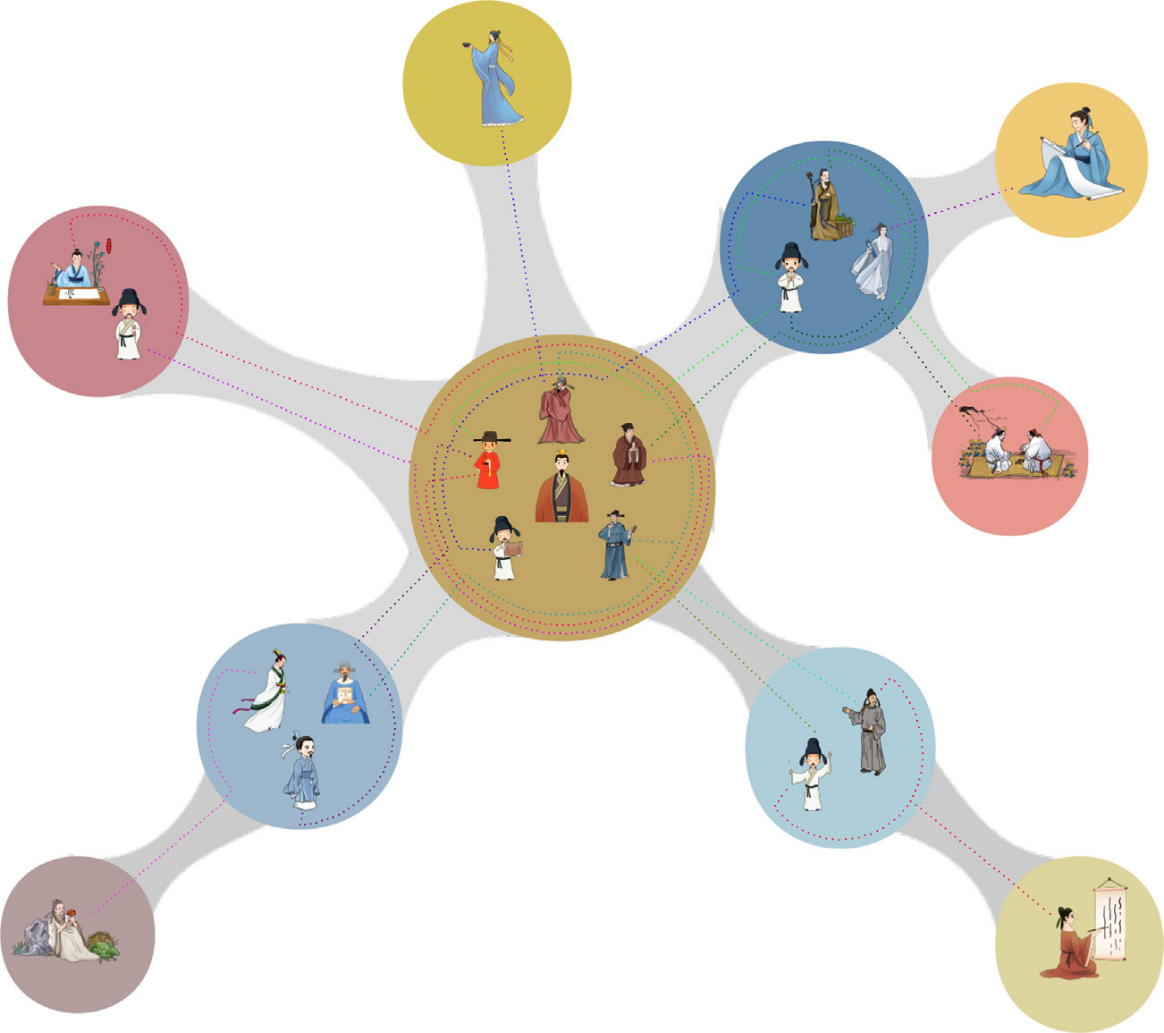
Demonstration of construction of social map. Circles and shadow lines denote the cities and their links; characters and dotted lines represent elites and social associations. The strength of a link between cities is the number of social associations of elites between these two cities.

In this dataset, an individual is defined as active between 20 years old and the index year. Therefore, an individual is supposed to be active during this time interval, otherwise inactive. A social map is presented by a weighted graph, G(V,A,W), in which each node $v_{i}\in V$ corresponds to a city, $a_{ij}\in A$ is an edge between $v_{i}$ and $v_{j}$. A weight of edge $w_{ij}\in W$ corresponds to the total number of social associations between two active elites, who locate in cities $v_{i}$ to $v_{j}$ respectively. The weight of the edge reflects the social strength between the two cities. For dynastic analysis, we generate the social map $G(t_{s},\;t_{e})$ performing further filtering, where $t_{s}$ and $t_{e}$, are the start and end year for each dynasty, respectively. Fig. 3 demonstrates the construction of social map.

For such a long history, the information we deal with may come with some uncertainty or noise, such as addresses and time stamps of individuals. To test the robustness of results, we estimate the influence of the uncertainty on the topological features. We randomly remove links between elites with probability p*,* and rebuild the social maps. The results are quite robust [1]. It verifies that this dataset is reliable.

## Ethics Statements

N/A.

## CRediT authorship contribution statement

**Xiong-Fei Jiang:** Conceptualization, Methodology, Supervision, Writing – review & editing. **Ling Bai:** Visualization, Software. **Na Zhao:** Visualization, Software. **Long Xiong:** Data curation, Writing – original draft.

## Declaration of Competing Interest

The authors declare that they have no known competing financial interests or personal relationships that could have appeared to influence the work reported in this paper.

## Data Availability

A dataset of dynamical social map in ancient China: 618 − 1644 (Original data) (Mendeley Data).

## Appendix Supplementary materials

Image, application 1
